# Preoperative prediction of lymphatic metastasis in rectal cancer using a fusion model based on multiparameter magnetic resonance imaging: a retrospective validation study

**DOI:** 10.3389/fonc.2026.1816420

**Published:** 2026-05-22

**Authors:** Peng Zheng, Donghao Xu, Kaiwen Chen, Zhekun Huang, Ziqi Zhang, Songbin Lin

**Affiliations:** 1 Department of Colorectal Surgery, Zhongshan Hospital, Fudan University, Shanghai, China; 2 Department of General Surgery, Zhongshan Hospital (Xiamen), Fudan University, Xiamen, China

**Keywords:** algorithms, deep learning, lymphatic metastasis, magnetic resonance imaging, rectal neoplasms

## Abstract

**Background:**

To validate an MRI-based deep learning algorithm for predicting lymphatic metastasis in rectal cancer (RC) and to construct an integrated fusion model combining imaging and clinicopathological factors to improve preoperative diagnostic performance.

**Methods:**

This study retrospectively included 127 patients with RC as a primary cohort and 33 patients from two other centers as an external validation cohort. All patients underwent radical resection for RC without preoperative radiotherapy or chemotherapy. Based on the MR images, the lymph nodes were interpreted by a previously constructed prediction algorithm and two radiologists independently. The clinical factors associated with lymph node metastasis (LNM) were screened by a logistic regression model and then combined with the prediction algorithm to construct a fusion model. The area under the receiver operating characteristic curve (AUC) and decision curve analysis (DCA) was used to evaluate the predictive power and clinical utility.

**Results:**

In the primary cohort, the prediction algorithm achieved an AUC of 0.760 (95% CI: 0.674–0.846), significantly outperforming the two radiologists [AUC: 0.665 and 0.676; interobserver kappa = 0.241]. Multivariate analysis identified a carcinoembryonic antigen (CEA) level >5 µg/L and poor differentiation as independent risk factors for LNM. The integrated fusion model (algorithm + CEA + differentiation) demonstrated superior performance with an AUC of 0.873 (95% CI: 0.804–0.972). In the external validation cohort, the fusion model showed promising diagnostic efficacy with an AUC of 0.838 (95% CI: 0.697–0.979). Decision curve analysis further confirmed that the fusion model provided higher clinical net benefit than default treatment strategies across a wide range of threshold probabilities.

**Conclusion:**

The preoperative fusion model significantly improves the accuracy of N-staging in rectal cancer. By outperforming manual interpretation and demonstrating encouraging preliminary generalizability in an external cohort, this model shows potential as a clinical aid for identifying patients who will benefit from neoadjuvant therapy, thereby facilitating personalized clinical decision-making and reducing interobserver variability.

## 1 Introduction

Colorectal cancer is one of the most common malignancies worldwide (1), and rectal cancer (RC) accounts for approximately one-third of those cases. Lymph node metastasis (LNM) is a common metastasis of RC that directly affects the prognosis of patients and is a crucial reference for decision-making in the treatment of RC (2). Clinical guidelines recommend that patients with cN+ RC should receive preoperative neoadjuvant chemoradiotherapy before surgery (3). Therefore, accurate assessment of LNM before treatment is critical.

Currently, pretreatment staging of RC mainly relies on manual interpretation of imaging examinations, especially MRI. However, this is a very time-consuming and laborious task that is strongly affected by the technical expertise of interpreters (4, 5). In previous studies, the reported accuracy of LNM according to MRI was about 55–70% (4, 6).

In recent years, deep learning (DL)-based radiomics has revolutionized the field of oncology and spearheaded novel approaches in the management of various cancers, including RC. Notably, DL radiomics to diagnose, prognosticate, and predict response to therapy in RC is gaining traction and proving to be promising (7, 8). While several models have been developed to evaluate LNM (9–16), most focus on T2-weighted and diffusion-weighted imaging (DWI) features and rely on the precise manual segmentation of individual lymph nodes—a process that is not only labor-intensive but also prone to significant interobserver variability, especially for nodes smaller than 3 mm (17). Critically, this specific ensemble—including T2WI, DWI, and five phases of dynamic contrast-enhanced (DCE) MRI—was identified as the optimal configuration in our previous foundational work through a systematic comparison of various sequence combinations (18). By shifting the analytical focus to the primary tumor region rather than individual nodes, our model captures a broader spectrum of tumor-driven microvascular and morphological features while bypassing meticulous nodal labeling. Furthermore, recognizing that image-based algorithms alone may not fully reflect the biological profile of the disease, we addressed a key research gap by integrating advanced DL features with routine clinicopathological biomarkers. The resulting fusion model aims to provide a more holistic and robust tool for preoperative risk stratification, ultimately facilitating more precise clinical decision-making.

The aim of this study was to validate our established MRI algorithm in a new consecutive patient set and develop a fusion model by integrating deep learning features with clinical biomarkers. This study intends to provide a more robust and clinically applicable tool for accurate preoperative N-staging in rectal cancer.

## 2 Methods

### 2.1 Study subjects

A primary cohort of patients with RC, diagnosed and treated at the Department of General Surgery, Zhongshan Hospital (Xiamen), Fudan University, between January 2022 and June 2024, was retrospectively continuously enrolled. To evaluate the model’s generalizability, an independent external validation cohort was established, comprising continuous patients from Xuhui District Central Hospital and Minhang District Central Hospital treated between January 2022 and December 2022. The same inclusion and exclusion criteria were applied to both cohorts to ensure consistency in data selection. The inclusion criteria were as follows: 1) pathologically confirmed rectal adenocarcinoma; 2) direct radical resection for RC; and 3) complete clinical, pathological, and preoperative MR image data. The exclusion criteria were as follows: 1) patients who could not undergo surgery because of surgical contraindications; patients who refused surgery for personal reasons; patients who were recommended to undergo radiotherapy and chemotherapy after multidisciplinary team (MDT) assessment; 2) patients who had a history of diagnosis and treatment of other malignant tumors. All patients signed informed consent forms. Postoperative adjuvant therapy and follow-up were completed according to the guidelines. This study was approved by the ethics committee of Zhongshan Hospital (B2024-058R).

### 2.2 Data collection

Clinicopathological data and multimodal MR images were retrieved from the institutional hospital information system (HIS) and picture archiving and communication system (PACS). Preoperative N-staging was independently evaluated by two senior radiologists (10 and 12 years of experience) who were blinded to all clinical information and pathological outcomes. To ensure consistency, a calibration session was conducted prior to the formal evaluation, and any discrepancies were resolved by consensus. Interobserver agreement was quantified using Cohen’s kappa analysis. For the primary tumor region, rough manual segmentation was performed on the portal venous phase of DCE-MRI using ITK-SNAP (version 3.6.0) as described in the **Supplementary Materials**. Magnetic resonance sequences were acquired using 1.5T and 3.0T scanners from multiple vendors (Siemens and United Imaging). To mitigate the impact of different scanning protocols and devices, all images underwent a standardized preprocessing pipeline, including B1 field inhomogeneity correction, voxel resampling to 1×1×1 mm³, and z-score intensity normalization. Detailed acquisition parameters are provided in **Supplementary Table 1**.

### 2.3 Prediction algorithm and fusion model construction

The prediction algorithm utilized in this study, termed MMENet (Multi-parametric Multi-scale EfficientNet), was developed based on our previous research (18). As illustrated in **Supplementary Figure 1**, the model employs a multi-branch convolutional neural network (CNN) architecture. The input consists of regions of interest (ROIs) from eight distinct MRI sequences (T2WI, two DWI, and five DCE phases). Each ROI is independently processed through five multi-scale feature extraction blocks, which utilize convolution kernels of varying sizes (3×3×3 and 5×5×5) to capture hierarchical features at different scales. A squeeze-and-excitation (SE) module is integrated into each block to adaptively re-weight channel-wise feature responses, enhancing the representation of tumor-driven microvascular patterns. The resulting feature maps are flattened into 32-dimensional feature vectors, concatenated, and passed through fully connected layers with a sigmoid activation to calculate the final LNM probability score.

To ensure the robustness of the deep learning backbone, the model was trained using the Adam optimizer with an initial learning rate of 1*10–^4^ and a batch size of 16. Regularization techniques, including ‘Dropout’ (rate = 0.5) and ‘Early Stopping’ (patience = 10), were implemented to prevent overfitting. Data augmentation (random rotation and flipping) was applied to the training set. Crucially, the primary cohort and external validation cohort in this study consist of newly enrolled patients, with no overlap with the training or testing datasets used in the original development of the MMENet algorithm.

For the construction of the fusion model, a logistic regression analysis was used to integrate the algorithm-derived probability with independent clinicopathological risk factors. To ensure the robustness of the results, the primary cohort was randomly partitioned into a training set and a testing set in an 8:2 ratio, and 5-fold cross-validation was implemented during the training phase to minimize optimism bias.

### 2.4 Statistical methods

Statistical analysis was performed using SPSS (version 25.0) and R software (version 4.5.2). Continuous variables are presented as mean ± standard deviation or median with interquartile range, depending on their distribution. Categorical variables are expressed as frequencies and percentages. Differences between groups were compared using the chi-square test or Fisher’s exact test. The predictive performance of the algorithm and radiologists was evaluated using sensitivity, specificity, accuracy, and the area under the receiver operating characteristic curve (AUC) with 95% confidence intervals (CI). Interobserver agreement between radiologists was quantified using Cohen’s kappa analysis. To evaluate clinical utility, decision curve analysis (DCA) was conducted. The DeLong test was employed to compare the AUC values between the model and radiologists. To evaluate the incremental value of the fusion model over the standalone algorithm, the net reclassification improvement (NRI) and integrated discrimination improvement (IDI) were calculated. Model calibration was assessed using the Hosmer-Lemeshow test. A two-sided *P* < 0.05 was considered statistically significant.

## 3 Results

### 3.1 Baseline characteristics and pathological data

**Figure 1:**
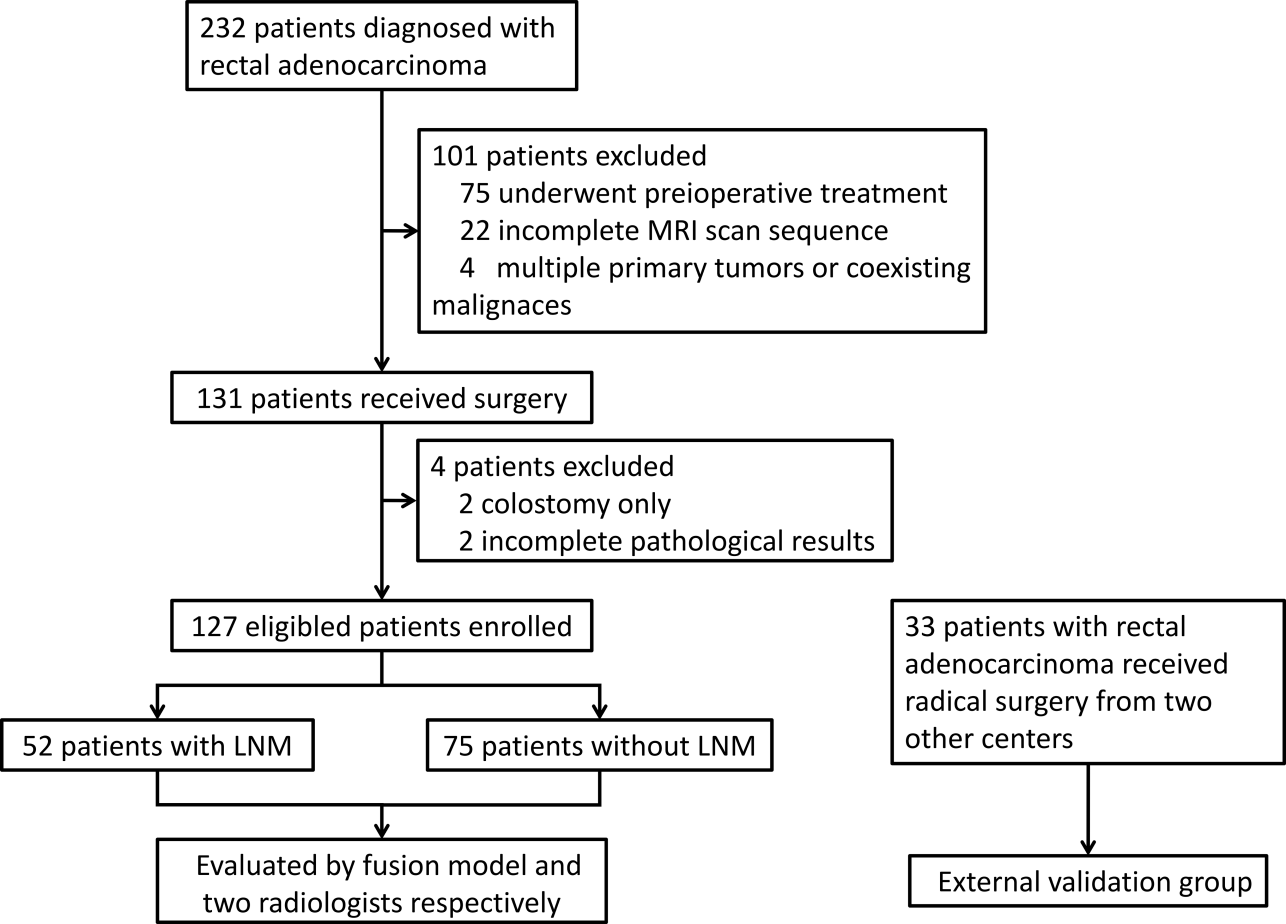
Flowchart of the study population selection. The diagram illustrates the step-by-step recruitment process for the primary cohort (*n* = 127) and the independent external validation cohort (*n* = 33). Notably, identical inclusion and exclusion criteria were rigorously applied to both cohorts to ensure data consistency and the reliability of the external validation. All patients were required to have pathologically confirmed rectal adenocarcinoma, have undergone direct radical resection, and possess complete preoperative multiparameter MR imaging data. Patients receiving neoadjuvant therapy or those with incomplete clinical information were excluded from both groups following the same protocol.

This study included a primary cohort of 127 patients with RC and an independent external validation cohort of 33 patients (**Figure 1**). Among the primary cohort, 75 patients (59.1%) were pathologically confirmed as LNM-negative (LNM-) and 52 (40.9%) as LNM-positive (LNM+). The baseline conditions and pathological data are shown in **Table 1**. In the primary cohort, patients with LNM demonstrated a significantly higher proportion of elevated preoperative CEA levels (>5 µg/L) compared to those without LNM (63.5% vs. 29.3%, p<0.001). Similarly, the LNM+ group showed a higher prevalence of poor histological differentiation compared to the LNM- group (30.8% vs. 10.7%, p=0.004).To evaluate the comparability of the datasets, we performed a statistical analysis of clinicopathological characteristics between the primary and external validation cohorts. As summarized in **Supplementary Table 2**, no significant differences were observed between two cohorts.

**Table 1:** Baseline characteristics and pathological data in primary cohort.

Baseline and pathological characteristics	LNM- (*n*=75)	LNM+(*n*=52)	*P* value
Gender [No. (%)]			0.153
Male	41 (54.7%)	35 (67.3%)	
Female	34 (45.3%)	17 (32.7%)	
Age [years, No. (%)]			0.990
> 60	52 (69.3%)	36 (69.2%)	
≤ 60	23 (30.7%)	16 (30.8%)	
American Society of Anesthesiologists (ASA) score [No. (%)]			0.882
I	52 (69.3%)	34 (65.3%)	
II	22 (29.3%)	17 (32.7%)	
III	1 (1.3%)	1 (1.9%)	
Preoperative CEA level [μg/L,No.(%)]			**< 0.001**
> 5	22 (29.3%)	33 (63.5%)	
≤ 5	53 (70.7%)	19 (36.5%)	
Preoperative CA19-9 level [U/ml, No. (%)]			0.848
> 37	8 (10.7%)	5 (9.6%)	
≤ 37	67 (89.3%)	47 (90.4%)	
Metastases [No. (%)]			0.141
Yes	10 (13.3%)	2 (3.8%)	
No	65 (86.7%)	50 (96.1%)	
Differentiation [No. (%)]			**0.004**
High/Moderate	67 (89.3%)	36 (69.2%)	
Poor	8 (10.7%)	16 (30.8%)	
pT stage [No. (%)]			0.198
T1-2	35 (46.7%)	16 (30.8%)	
T3-4	40 (53.3%)	36 (69.2%)	
lymph nodes harvested [M(Q1-Q3)]	16 (13-18)	15 (14-18)	0.761
Neurovascular involvement [No. (%)]			**< 0.001**
Yes	24 (32.0%)	36 (69.2%)	
No	51 (68.0%)	16 (30.8%)	
RAS status [No. (%)]			0.858
Wild-type	48 (64.0%)	29 (55.8%)	
Mutant	27 (36.0%)	23 (44.2%)	
BRAF status [No. (%)]			0.927
Wild-type	73 (97.4%)	48 (92.3%)	
Mutant	2 (2.6%)	4 (7.7%)	
MMR status [No. (%)]			0.656
pMMR	74 (98.7%)	50 (96.1%)	
dMMR	1 (1.3%)	2 (3.8%)	

LNM, Lymph node metastases; MMR, Mismatch repair. Bold values indicated statistically significant p values.

### 3.2 Diagnostic performance of the prediction algorithm and radiologists

**Figure 2:**
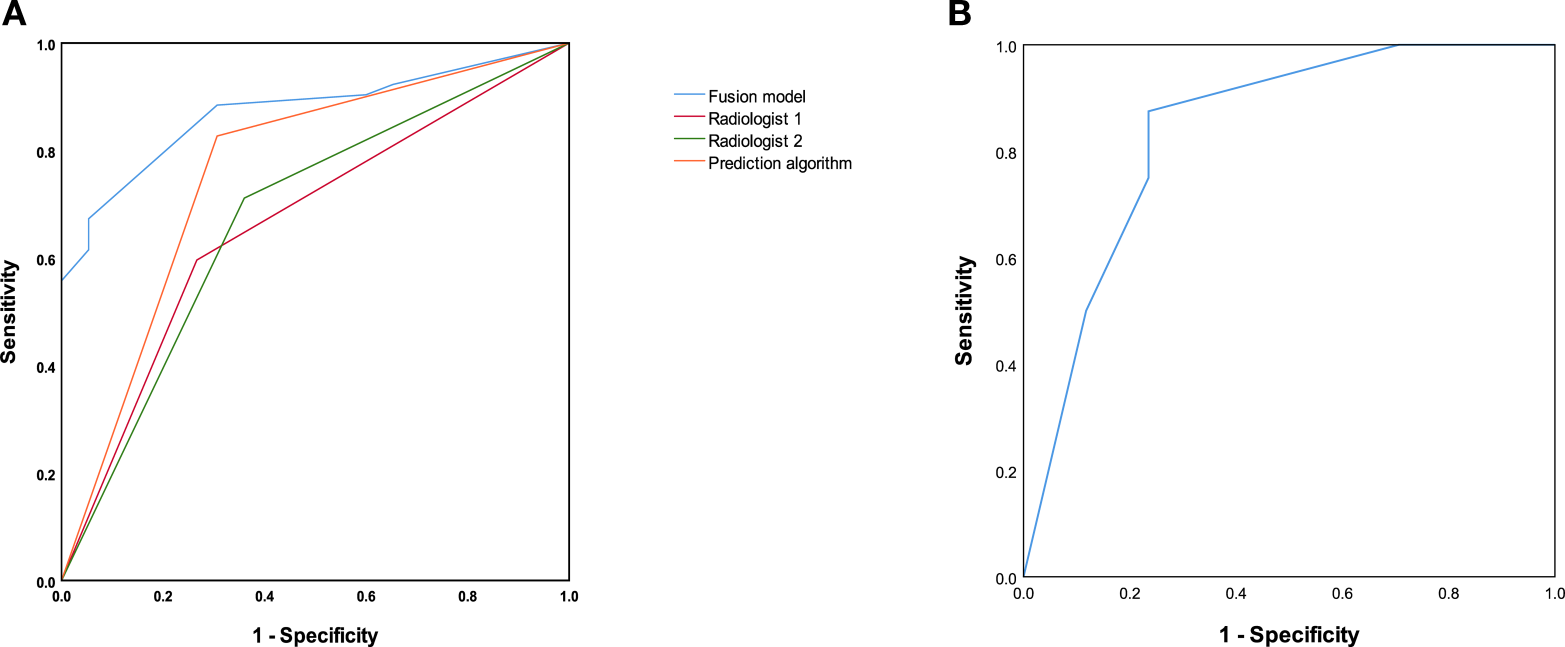
Receiver operating characteristic (ROC) curves for the prediction algorithm, fusion model, and manual interpretation. **(A)** in the primary cohort (*n* = 127), the fusion model (blue line), which integrates the deep learning algorithm with CEA levels and tumor differentiation, achieved the highest area under the curve (AUC = 0.873), significantly outperforming the standalone prediction algorithm and the two experienced radiologists. **(B)** in the independent external validation cohort (*n* = 33), the fusion model maintained robust diagnostic efficacy (AUC = 0.838), demonstrating its stability across different scanning protocols and institutions.

The prediction algorithm and the two radiologists independently evaluated the status of LNM based on MR images. The diagnostic metrics, including sensitivity, specificity, positive predictive value (PPV), negative predictive value (NPV), and accuracy, are summarized in **Table 2**. The prediction algorithm achieved an AUC of 0.760 (95% CI: 0.674–0.846), which was higher than those of the two experienced radiologists [0.665 (95% CI: 0.657–0.762) and 0.676 (95% CI: 0.580–0.771), respectively; **Figure 2]**. However, the DeLong test indicated that these differences did not reach statistical significance (p = 0.091 and 0.116, respectively). Furthermore, the algorithm demonstrated superior sensitivity (82.7%) compared to the radiologists (59.6% and 71.2%). Cohen’s kappa analysis yielded a value of 0.241, indicating only fair agreement between the two radiologists. This discrepancy suggests a notable instability in manual interpretation, highlighting the clinical value of the more objective and consistent assessment provided by the algorithm.

**Table 2:** Comparison between prediction algorithm and two radiologists.

Golden standard and predictive efficacy		Radiologist 1		Radiologist 2		Prediction algorithm	
		N0	N+	N0	N+	N0	N+
Pathological data	pN0 (*n* = 75)	51	24	48	27	52	23
	pN1/2 (*n* = 52)	21	31	15	37	9	43
Predictive value	Sensitivity	59.6% (46.0%–71.9%)		71.2% (57.9%–81.5%)		82.7% (70.6%–90.5%)	
	Specificity	68.0% (56.8%–77.5%)		64.0% (52.7%–73.9%)		69.3% (58.1%–78.7%)	
	PPV	60.8% (43.3%–68.6%)		57.8% (45.6%–69.1%)		65.2% (53.1%–75.5%)	
	NPV	70.8% (59.5%–80.0%)		76.2% (64.4%–85.0%)		85.2% (74.3%–92.0%)	
	Accuracy	64.5% (55.9%–72.3%)		66.9% (58.3%–74.5%)		74.8% (66.6%–81.6%)	

NPV, Negative predictive value; PPV, Positive predictive value.

### 3.3 Construction and validation of the integrated fusion model

Clinicopathological factors associated with LNM were first screened using univariate and multivariate analyses. Variables for the multivariate model were selected based on their statistical significance in univariate analysis and their availability in the preoperative clinical setting. Consequently, although neurovascular involvement was significant in the baseline comparison, it was excluded from the multivariate analysis as it is a postoperative pathological feature. The results of multivariate analyses identified a CEA level >5 µg/L and poor tumor differentiation as independent risk factors for LNM (**Table 3**). Then, a logistic regression model was employed to integrate the prediction algorithm with these two biomarkers to construct a fusion model. In the primary cohort, the fusion model achieved a significantly improved AUC of 0.873 (95% CI: 0.804–0.972; **Figure 2**). DeLong test confirmed that the fusion model significantly outperformed both radiologists (p < 0.001 and p < 0.001, respectively).

**Table 3:** Univariate and multivariate analysis of LNM.

Variates	Univariate		Multivariate	
	OR (95% CI)	p value	OR (95% CI)	p value
Gender: Male vs. Female	0.59(0.28-1.22)	0.155		
Age (years): >60 vs. ≤60	1.01(0.47-2.16)	0.990		
CEA (μg/L): >5 vs. ≤5	4.18(1.97-8.88)	**< 0.001**	8.60 (2.93-25.2)	**< 0.001**
CA19-9 (U/ml): >37 vs. ≤37	1.12(0.35-3.65)	0.848		
Differentiation: Poor vs. High-Moderate	3.72(1.45-9.53)	**0.006**	3.86(1.12-14.3)	**0.006**
RAS status: Mutant vs. Wild-type	1.41(0.69-2.90)	0.351		
BRAF status: Mutant vs. Wild-type	3.04(0.54-17.2)	0.209		
MMR status: dMMR vs pMMR	2.06(0.26-12.1)	0.775		
Prediction algorithm: LNM+ vs LNM-	10.8(4.53-25.8)	**< 0.001**	18.2(6.02-55.3)	**< 0.001**

CEA, Carcinoembryonic antigen; OR, Odds ratio; CI, Confidence interval; LNM, Lymph node metastases; MMR, Mismatch repair.

Bold values indicated statistically significant p values.

To enhance clinical interpretability, the model’s performance was evaluated at two distinct threshold points. At the point of maximizing the Youden index, the fusion model demonstrated an accuracy of 81.1% and a specificity of 94.7%. However, considering the clinical priority of avoiding under-treatment in rectal cancer, we also evaluated the model at a high-sensitivity threshold. At this point, the sensitivity reached 92.3%, effectively identifying the vast majority of patients who would benefit from neoadjuvant chemoradiotherapy. To facilitate clinical application, a nomogram was constructed based on the logistic regression model (**Supplementary Figure 3**).

**Figure 3:**
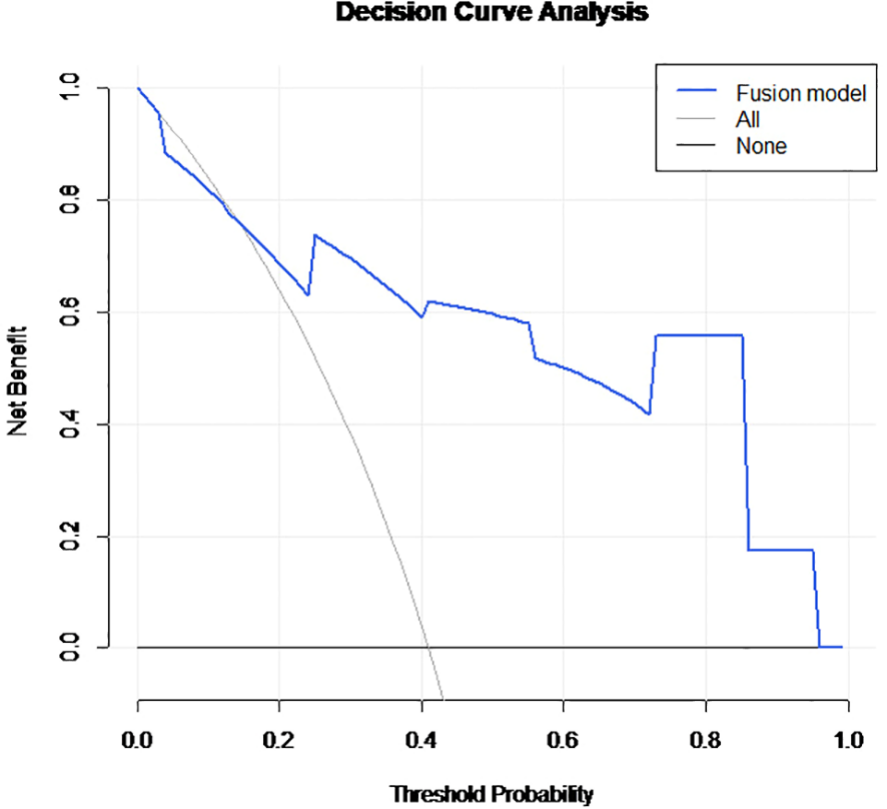
Decision curve analysis (DCA) for the fusion model. The DCA curve demonstrates the clinical net benefit of the fusion model across a wide range of threshold probabilities. The blue line (fusion model) consistently remains above the “treat-all” (gray line) and “treat-none” (horizontal black line) strategies, indicating superior clinical utility in identifying lymph node metastasis.

The performance of the fusion model was further evaluated in the independent external validation cohort (*n* = 33), where it maintained a high diagnostic efficacy with an AUC of 0.838 (95% CI: 0.697–0.979; **Figure 2**). Finally, DCA was performed to evaluate clinical utility. The DCA indicated that the fusion model provided a higher net benefit compared to both the ‘treat-all’ and ‘treat-none’ strategies across a wide range of threshold probabilities (**Figure 3**), highlighting its potential for optimizing preoperative decision-making.

The fusion model demonstrated excellent calibration (Hosmer-Lemeshow test, p = 0.614). Furthermore, compared to the standalone algorithm, the fusion model yielded a significant NRI of 0.612 (95% CI: 0.376 - 0.850, p<0.001) and IDI of 1.934 (95% CI: 1.459 - 2.386, p<0.001), indicating its superior ability to accurately reclassify patients’ nodal status.

## 4 Discussion

This study successfully validated a multiparameter MRI-based deep learning algorithm and established a clinically integrated fusion model for predicting LNM in RC. Our results demonstrate that the fusion model, which combines imaging features with CEA and tumor differentiation, achieved superior diagnostic performance with an AUC of 0.873. Crucially, the model maintained satisfactory performance in an independent external validation cohort (AUC: 0.838). This transition from a purely image-based approach to a multi-dimensional fusion model offers a potential framework for a standardized tool for preoperative N-staging.

The primary aim of this work was to transition from a previously established image-based prediction algorithm to a more comprehensive, clinically integrated fusion model while validating its diagnostic efficacy and generalizability in a new patient set. By integrating readily obtainable clinicopathological biomarkers with advanced deep learning features, the study intended to provide a more robust and clinically applicable tool to assist in the selection of candidates for neoadjuvant chemoradiotherapy.

Currently, mainstream clinical guidelines recommend MRI as the gold standard for local staging of rectal cancer (3, 19). However, the diagnostic ability of MRI for LNM is limited, with a sensitivity of 58–77% and a specificity of 62–84% (9, 20), which are similar to those of computed tomography (CT) and ultrasound examinations (21). The reason is that, although the size of the lymph nodes is still the main criterion for interpretation, approximately 30–50% of the metastatic lymph nodes are ≤5 mm on MR images. In addition, LNM is more common in T3/4 patients without enlargement (22). Conversely, approximately 25% of negative lymph nodes are overestimated, which is generally caused by inflammation/fibrosis (23). This discrepancy frequently results in incorrect N-staging, which can lead to suboptimal treatment selection. Our fusion model was specifically designed to overcome these challenges by capturing multi-dimensional features that transcend human visual assessment.

Recently, several studies have explored the potential of computer-aided systems for LNM prediction in RC (15, 24, 25). Li et al. combined clinical risk factors and radiomics features to construct a prediction model, and the optimal AUC under different combinations reached 0.7606 (13). Zhou et al. integrated the interpretation results of radiologists on top of a multiparameter MRI radiomics model to evaluate the condition of lymph nodes in patients with RC after chemoradiotherapy. The results revealed that the positive predictive value of the integrated model was 93.7% and reached 100% in the ypT1–2 subgroup (14). However, a major limitation of these existing models is their heavy reliance on the precise manual segmentation of individual lymph nodes. This process is not only time-consuming but also prone to significant interobserver variability, particularly for metastatic nodes smaller than 5 mm that often appear morphologically normal on MRI. Instead of obsessing over individual nodes, we trained the system to analyze the primary tumor region itself. The biological underpinning of our model’s predictive power lies in the comprehensive characterization of the tumor microenvironment (TME). While traditional staging relies on the morphological enlargement of lymph nodes, LNM is fundamentally driven by the biological aggressiveness of the primary tumor. By integrating eight MRI sequences, the model captures a multi-dimensional ‘radiomic phenotype’: T2-weighted imaging depicts morphological heterogeneity and invasive borders; DWI reflects increased cell density and restricted diffusion; and DCE-MRI phases quantify tumor-driven angiogenesis and microvascular permeability. These ‘latent’ aggressive traits are often indicative of a high metastatic potential (26, 27) that precedes macroscopic nodal enlargement, explaining why our tumor-centered approach outperforms manual node-counting.

The superiority of the fusion model is further reflected in its clinical utility. Multivariate analysis identified CEA level >5 µg/L and poor differentiation as independent risk factors, which align with prior evidence that high tumor burden and aggressive biological behavior are correlated with LNM (28). In future studies, the integration of pathomics or genetic status may further improve the prediction performance for LNM. Actually, we have tried hotspot gene mutations (RAS and BRAF) in colorectal cancer, but the results indicated no improvements.

To evaluate the clinical net benefit, we performed a DCA, which demonstrated that the fusion model provided a higher clinical net benefit than default “treat-all” or “treat-none” strategies across a wide range of threshold probabilities. In clinical practice, our fusion model provides a standardized decision-support tool for Multidisciplinary Teams (MDT). To bridge the gap between AI outputs and clinical action, we developed a nomogram that allows for individualized risk estimation. During MDT sessions, the model’s high-sensitivity threshold (92.3%) can be prioritized to identify patients who would most benefit from neoadjuvant chemoradiotherapy (nCRT), thereby reducing the risk of under-treatment. Conversely, for patients categorized as low-risk by both the algorithm and clinicopathological markers, the model offers the potential to spare them from the toxicity of unnecessary radiation. This objective scoring system effectively mitigates the fair inter-observer agreement (kappa = 0.241) observed among manual interpretations, ensuring more consistent treatment pathways.

A notorious challenge in radiomics-based research is the lack of reproducibility and generalizability, as heterogeneous scanning devices, varying acquisition protocols, and subjective ROI segmentation methods significantly confound the extracted features (29, 30). Our study was specifically designed to circumvent these hurdles by streamlining ROI segmentation and enhancing model compatibility with multi-source, variable-quality MR images. Compared to previously reported algorithms, our approach offers two distinct advantages. First, our model eliminates the requirement for meticulous manual labeling of individual target lymph nodes. We not only significantly reduce the clinical workload but also mitigate the impact of human-factor variability on diagnostic outcomes. While this architecture precludes the granular determination of the exact number and anatomical location of metastatic nodes, it substantially improves interpretation efficiency and facilitates the large-scale clinical promotion of the algorithm. Second, whereas traditional algorithms are often restricted to T2-weighted and DWI sequences, our algorithm achieves superior stability through the integration of eight distinct MRI sequences. Consequently, the model demonstrates remarkable robustness in real-world clinical settings, maintaining high diagnostic performance even when image quality is suboptimal. This resilience is evidenced by our inclusion of cases with relatively thick slices—up to 5.5 mm for sagittal T2 and 7 mm for DWI—which would typically compromise the accuracy of less robust radiomics models.

Despite the promising results, several limitations warrant acknowledgment. First, our study population was restricted to a “surgical-only” cohort, which introduces a potential spectrum-selection bias. By excluding patients who received neoadjuvant chemoradiotherapy (nCRT), the model’s performance was validated in a population that might represent a relatively lower clinical N-stage spectrum compared to the general RC population. However, this design was necessary to maintain the integrity of the pathological ground truth; nCRT often induces nodal regression, making it impossible to determine the true pretreatment lymphatic status via post-surgical pathology. Consequently, while the model demonstrates high accuracy for patients undergoing direct surgery, its transportability to patients already designated for nCRT requires further investigation. Future studies should focus on evaluating the model’s utility in predicting nodal response in post-nCRT settings. Second, although the external validation cohort provided essential support for generalizability, the sample size remains relatively limited. Larger multi-center prospective studies are needed to further refine the model. Finally, this study focused on the independent validation of the algorithm and fusion model. Future research should explore a reader-AI collaboration scenario to evaluate how the algorithm’s objective score can directly assist radiologists in real-world clinical workflows and further mitigate the observed inter-observer variability.

## 5 Conclusion

In conclusion, our study validated a multiparameter MRI-based deep learning algorithm and established a fusion model for predicting lymphatic metastasis in rectal cancer. By synergizing image-extracted features with clinicopathological biomarkers (CEA and differentiation), the fusion model achieved superior diagnostic accuracy and clinical net benefit compared to conventional radiologist assessment. The performance observed in the external validation cohort suggests the model’s potential for future clinical implementation. This integrated approach presents a promising framework for preoperative risk stratification, which may assist in optimizing the selection of candidates for neoadjuvant chemoradiotherapy and contribute to the advancement of precision medicine in rectal cancer management.

## Data Availability

The original contributions presented in the study are included in the article/**Supplementary Material**. Further inquiries can be directed to the corresponding author.
