# Supplementary material for: Preoperative prediction of lymphatic metastasis in rectal cancer using a fusion model based on multiparameter magnetic resonance imaging: a retrospective validation study
Source: Front Oncol. 2026 May 22;16:1816420. doi: 10.3389/fonc.2026.1816420 (PMC13236564; doi:10.3389/fonc.2026.1816420)
Supplement: Supplementary file 1 [file DataSheet1.docx]

**Supplementary materials**

**Collection, delineation and standardization of MRI sequences**

MRI sequences were acquired using 1.5-Tesla systems (uMR 560, United Imaging Healthcare; uMR 680, United Imaging Healthcare; Aera, Siemens Healthcare; Avanto, Siemens Healthcare) and 3.0-Tesla systems (uMR 770, United Imaging Healthcare; uMR 790, United Imaging Healthcare; Verio, Siemens Healthcare) with a 12/16/18 channel phased-array body coil. Each case contained 10 sequences, and the image acquisition requirements are presented in **Supplementary Table 1**. The accessible MRI sequences included: (1) Diffusion weighted imaging (DWI): single-shot spin-echo echo-planar imaging at b-values of 0 and 1000 sec/mm with automatically calculated apparent diffusion coefficient (ADC) maps based on these b-values; (2) T2-Weighted Imaging (T2WI): sagittal T2-weighted turbo spin-echo pulse imaging; and (3) DCE-MRI: dynamic three-dimensional T1-weighted gradient-echo volumetric-interpolated breath-hold examination sequence, obtaining axial precontrast phase, arterial phase (20–30 s), portal venous phase (70–80 s), coronal delayed phase, and sagittal delayed phase (about 180 s) after the bolus injection of gadopentetate dimeglumine (Magnevist; Bayer Schering Pharma AG, Berlin, Germany).

Because the MRI sequences are already aligned during the MRI scan, radiologists only need to roughly outline the tumor region on the portal venous-phase sequence, with the corresponding tumor regions in the remaining sequences inferred from this outline. One radiologist delineated the tumor contours with ITK-SNAP (version 3.6.0; <http://www.itksnap.org).>

To standardize image data from different scanners, a consistent processing pipeline was implemented, comprising resampling, cropping, resizing, and normalization. First, the MRI images were resampled to achieve a uniform voxel spacing of 1 × 1 × 1 mm using third-order spline interpolation. Subsequently, the images were cropped to a tumor-centered volume of 128 × 128 × 64 voxels on the basis of tumor annotations in the portal venous-phase sequence. The cropped volume was then resized to dimensions of 64 × 64 × 64 voxels. Finally, each image underwent individual z-score normalization on the basis of the mean and standard deviation of the intensity values.

**Supplementary Table 1.** MR image acquisition requirements

| No | Sequences | Slice thickness (mm) |
| --- | --- | --- |
| 1 | DWI(B=800/1000) | 6-6.5 mm |
| 2 | DWI ADC | 6-6.5 mm |
| 3 | T2 Sagittal | 4 mm |
| 4 | T2 Axial | 4 mm |
| 5 | T1 Axial precontrast phase | 3-4 mm |
| 6 | T1 Axial Arterial phase | 3-4 mm |
| 7 | T1 Axial Portal phase | 3-4 mm |
| 8 | T1 Sagittal | 3-4 mm |
| 9 | T1 Coronal | 3-4 mm |
| 10 | T1 Axial delayed phase | 3-4 mm |

**Supplementary Table 2**. Comparison of baseline clinicopathological characteristics between the primary cohort and the external validation cohort.

|  | Primary cohort  (*n*=127) | external validation cohort (*n*=33) | *P* value |
| --- | --- | --- | --- |
| Gender [No. (%)] |  |  | 0.472 |
| Male | 76 (59.8%) | 22 (66.7%) |  |
| Female | 51 (40.2%) | 11 (33.3%) |  |
| Age [years, No. (%)] |  |  | 0.203 |
| > 60 | 88 (69.3%) | 19 (57.6%) |  |
| ≤ 60 | 39 (30.7%) | 14 (42.4%) |  |
| Preoperative CEA level [ug/L，No.(%)] |  |  | 0.822 |
| > 5 | 55 (43.3%) | 15 (45.5%) |  |
| ≤ 5 | 72 (56.7%) | 18 (54.5%) |  |
| Preoperative CA19-9 level [U/ml, No. (%)] |  |  | 0.424 |
| > 37 | 13 (10.2%) | 5 (15.1%) |  |
| ≤ 37 | 114 (89.8%) | 28 (84.8%) |  |
| Differentiation [No. (%)] |  |  | 0.925 |
| High/Moderate | 103 (81.1%) | 27 (81.8%) |  |
| Poor | 24 (18.9%) | 6 (18.2%) |  |
| pT stage [No. (%)] |  |  | 0.091 |
| T1-2 | 51 (40.2%) | 8 (24.2%) |  |
| T3-4 | 76 (59.8%) | 25 (75.8%) |  |
| pN stage [No. (%)] |  |  | 0.277 |
| N0 | 75 (59.1%) | 16 (48.5%) |  |
| N1-2 | 52 (40.9%) | 17 (51.5%) |  |


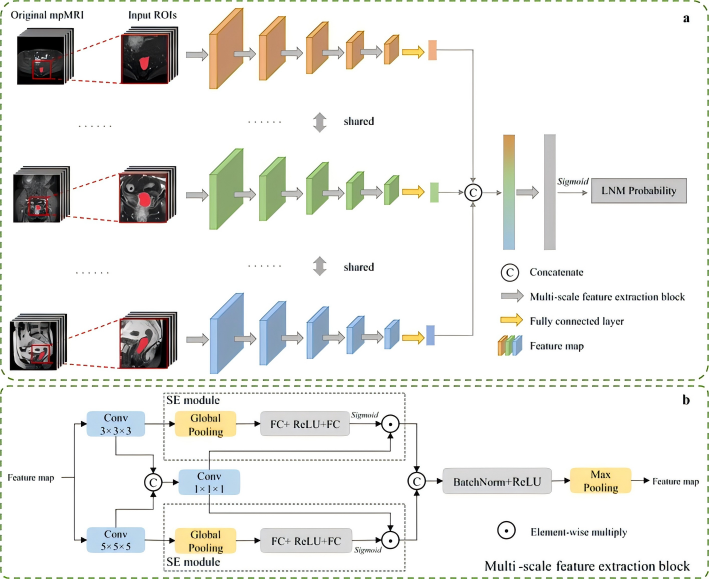


**Supplementary Figure 1.** **Pipeline of the deep learning-based prediction algorithm**. (A) Image preprocessing and deep network architecture; (B) Multi-scale feature extraction block.

Abbreviations: ROI, region of interest; FC, fully connected layer; Conv, convolutional layer; LNM, lymph node metastasis.


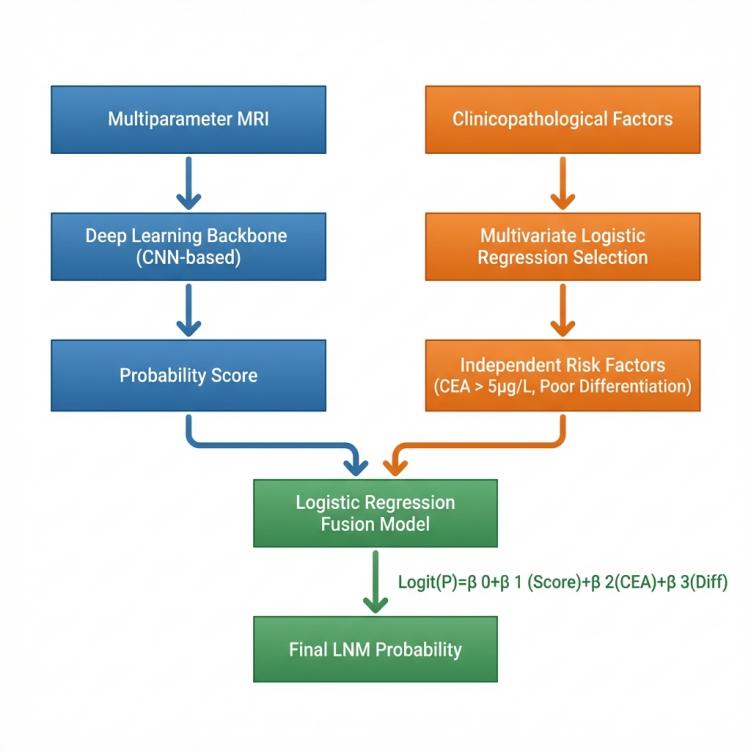


**Supplementary Figure 2. Architecture of the integrated fusion model.** This flowchart demonstrates the construction of the fusion model. The quantitative probability score was derived from the deep learning backbone. Multivariate logistic regression analysis identified CEA level and differentiation as independent risk factors, which were subsequently included in the fusion framework alongside the algorithm score. The final model is expressed via a logistic regression equation.


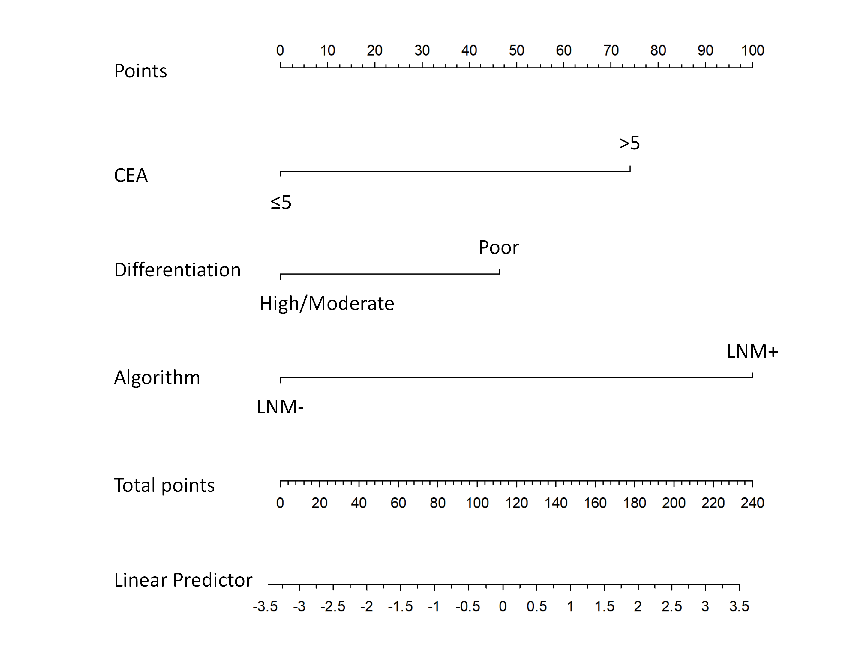


**Supplementary Figure 3.** Nomogram for the preoperative prediction of lymphatic metastasis (LNM) in rectal cancer. The nomogram was developed based on the integrated fusion model, incorporating the MRI-based deep learning algorithm score, preoperative CEA level (µg/L), and histological differentiation. This tool aims to assist multidisciplinary teams (MDT) in preoperative risk stratification and clinical decision-making.
